# DTL versus skin electrodes in recording of multifocal pattern electroretinogram and multifocal photopic negative response

**DOI:** 10.1007/s10633-025-10014-5

**Published:** 2025-04-09

**Authors:** Henrike Marie Nowitzki, Michael B. Hoffmann, Khaldoon O. Al-Nosairy

**Affiliations:** 1 Section for Clinical and Experimental Sensory Physiology, Ophthalmology, Otto-Von-Guericke-University Magdeburg, Leipziger Str. 44, 39120 Magdeburg, Germany; 2 Center for Behavioral Brain Sciences, Magdeburg, Germany

**Keywords:** mfPERG, mfERG_PhNR_, PhNR, ERG, Skin electrode, DTL electrode, SNR

## Abstract

**Objective:**

To compare the photopic negative response of the multifocal ERG (mfERG_PhNR_) and the multifocal pattern electroretinogram (mfPERG) using DTL electrode (E_DTL_) vs skin electrode (E_SKIN_) in healthy young and old adults.

**Methods:**

Ten “Young” [20–27 years] and eight “Old” [60–72 years] participants took part in this study. The electrophysiological responses were recorded binocularly using E_DTL_ and E_SKIN_. 5-way ANOVAs were applied to investigate the following factors on mfERG_PhNR_: i) ELECTRODE, ii) DILATATION, iii) AGE, iv) EYE, and v) ECCENTRICITY. For mfPERG, the same factors, except dilatation, were investigated applying 4-way ANOVAs. These were conducted for amplitude and peak time of different components as well as signal-to-noise-ratio (SNR).

**Results:**

Amplitudes of mfERG_PhNR_ [mfPERG]-based E_SKIN_ recording were reduced to 32–38% [37–38%] compared to E_DTL_, $p < 0.001$. This corresponded to SNR reduction to 80% [60%], $p < 0.001$. E_SKIN_ based responses had shorter peak times, by 0.2–0.5 ms for N1 and P1, $p < 0.05$, [P1: 1.5 ms, $p < 0.001$]. Both age groups had comparable amplitudes and SNRs, but Young had shorter peak times, by 1.5–2.2 ms for N1 and P1, $p < 0.05$ [3.7–4.2 ms for N1, P1, N2, $p < 0.05$]. Compared to dilated recordings, undilated mfERG_PhNR_ amplitudes were reduced to 47–87%, $p < 0.01$, and peak times were delayed by 2.0–11.8 ms, $p < 0.001$.

**Conclusions:**

mfPERG & mfERG_PhNR_ traces were similar for E_DTL_ and E_SKIN_. However, for skin electrodes, amplitudes and SNRs were lower and peak times shorter. E_SKIN_ thus seem to be a viable alternative in patients in whom the use of corneal electrodes is precluded, e.g., children and disabled patients, but at the expense of SNR and with reference to E_SKIN_ normative data.

## Introduction

In clinical practice, the objective assessment of the functional integrity of the retina with the electroretinogram (ERG) is indispensable to detect, rule out and follow-up visual system abnormalities and diseases. The full-field ERG allows for the assessment of retinal function including photoreceptor and bipolar cell integrity (a- and b-wave), while the pattern ERG and the photopic negative response (PhNR) of the ERG allow for the assessment of retinal ganglion cell integrity [1]. However, the application of ERG recordings can be challenged by the use of corneal electrodes, e.g., children, intolerance to corneal electrodes, patient’s refusal, or oculo-vagal reflex. To overcome such hurdles, the viability of skin electrodes has previously been tested, e.g., for the full-field ERG [2–7], the photopic negative response (PhNR) [8–10], and the pattern ERG (PERG) [11, 12]. However, these conventional ERG recordings reflect retinal function in a non-spatially resolved manner. A spatial resolved account of retinal function and dysfunction is expected to be of benefit in both clinical and research applications, e.g., in the assessment of hydroxychloroquine toxicity [13] or glaucoma damage [14].

With multifocal stimulation, visual function can be assessed in a spatially resolved manner [15]. Combined with the ERG, this resulted in the multifocal ERG (mfERG), which is now a routine tool in the clinical electrophysiology of vision for the assessment of the visual field topography of retinal function [16]. In addition, other ERG types have been combined with the multifocal technique, resulting in the multifocal Photopic Negative Response of ERG (mfERG_PhNR_) [14, 17] and the multifocal pattern electroretinogram (mfPERG) [18–20]. These ERG types assist the assessment of retinal dysfunction in e.g., glaucoma and multiple sclerosis [14, 17, 19, 20]. Importantly, the use of non-corneal electrodes for multifocal electroretinography is expected to make them available for a wider range of applications and patient groups. While the viability of skin electrode recordings has been assessed previously for the mfERG [21], there is currently still a lack of such investigations for the mfERG_PhNR_ and mfPERG.

In the present study we aimed to fill this gap and compared mfPERG and mfERG_PhNR_ for skin vs corneal, i.e. DTL [22], electrodes.

## Methods

### Participants

This prospective cross-sectional study followed the tenets of the declaration of Helsinki and was approved by the ethics committee of the Otto-von-Guericke University of Magdeburg, Germany. Participants gave their written consent prior to the study.

Two groups were recruited: i) Ten “Young” participants with an age range of 20–27 [years] and a mean ± SD of 22.5 ± 2.1 [years] vs ii) Eight “Old” participants within age range of 60–72 [years] and a mean ± SD 66.1 ± 4.2 [years]. Both groups had refractive errors <  ± 4 diopters, best corrected visual acuity (BCVA) in both groups was < 0.1 in logMAR (logarithm of the minimum angle of resolution), mean ± SD in Young − 0.15 ± 0.09 and in Old − 0.11 ± 0.05 (no significant group difference, $p = 0.190$).

All participants underwent a full eye examination to exclude possible abnormalities, e.g., glaucoma. Visual acuity was tested with the EDTRS chart (Early Treatment Diabetic Retinopathy Study) at 4 m and proper near refraction for the ERG viewing distance, 36 cm. Normal visual function was assessed using visual field testing with the Swedish Interactive Threshold Algorithm 24-2 protocol (SITA-Fast) of the Humphrey Field Analyzer 3 (Carl Zeiss Meditec AG, Jena, Germany).

### Electrophysiological recordings

**Fig. 1:**
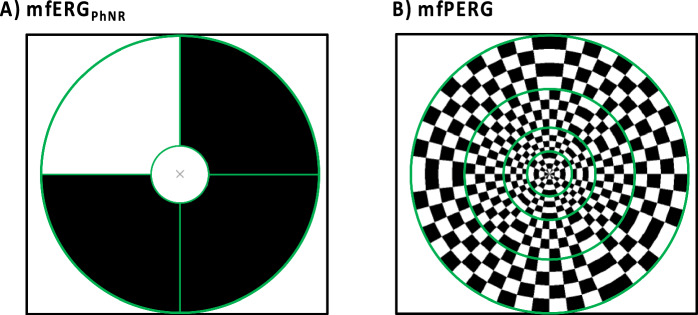
**(A)** The mfERG_PhNR_ stimulus spanning 2 eccentricities with 5 stimulated fields (snapshot with 3 peripheral locations “off”, and the central “on”) and **(B)** mfPERG stimulus spanning 4 eccentricities with a total of 36 stimulated visual field patches (4 × 4 checkerboards). Green lines serve to clarify the eccentricities/borders and are not seen by the participant

Binocular mfPERG and mfERG_PhNR_ are two types of ERG recordings, see Fig. 1 for stimulus and Table 1 for further details. Each was recorded simultaneously with a DTL electrode (DTL Electrode ERG, Unimed electrode Supplies, Ltd, UK) [22] placed across the cornea along the lower lid and a gold-cup skin electrode (10 mm diameter Golden EEG Cup Electrodes, Natus Manufacturing Limited, Ireland) placed on the lower lid 5 mm below the lid margin. The DTL electrode-based and the skin electrode-based recordings are referred to as “E_DTL_” and “E_SKIN_”, respectively, in the present study. Both active DTL and skin electrodes were referenced to a gold-cup skin electrode at the ipsilateral canthus. Gold-cup electrodes were filled with conductive paste (Ten20, WEAVER and Company, USA) and attached to the skin after cleaning with paste (skinPure, NIHON KODEN Corporation, Tokyo, Japan) to reduce the resistance of the skin to < 5 kOhm.

**Table 1:** Multifocal retinal function recordings

	mfERG_PhNR_^1^	mfPERG^2^
Stimulus delivery and recording	VERIS Science^3^	
Display device	monochrome CRT-monitor^4^ with 75 Hz frame rate & achromatic stimuli	
Viewing distance	36 cm	
Room light	dimly lit	
Eye	binocular recording	
Stimulated visual field (diameter)	46°	45°
# of visual field patches	5	36
Eccentricities	Central ring: 4.8° Outer ring (4 fields): 23.1°	Ring1: 0.0–3.6, ring2: 3.6–7.6 Ring3: 7.6–14.3; ring4: 14.3–22.7°
Stimulus type	Bright flash stimulus (0) state: no flash (1) state: flash 9 interleaving frames	4 × 4 checkerboard pattern reversal (pr)^5^ (1) state: Pattern 1 (2) state: Pattern 2 2 frames per state [23]
Traces	Figure 1A	Figure 1B
Mean luminance [cd/m^2^]	104 min 7; max 200	56 min: 5; max: 130
m-sequence; step duration	2^9^–1; 13.3 ms [17]	2^14^–1; 26.6 ms [23]
Recording settings	The signals amplified by 100K^6^ band pass filtered 3–300 Hz & digitized at 1200 Hz. Traces were then digitally filtered (3–45 Hz)^7^	
Response analysis	1st order kernel	1st slice of 2nd order kernel
No. of segments^8^	16	32
Repetitions	3 times, undilated pupils vs 3 times, dilated pupils^9^	3 times, undilated pupils
SNR^10^	Signal: 0–100 ms; Noise: 700–800 ms	Signal: 0–100 ms; Noise: 300–400 ms
Components, origin, nomenclature	N1, 1st negativity: “cone-photoreceptors/bipolars”^11^ [24, 25] P1, 1st positivity: cone bipolar cells, horizontal cells [26] N2†, 2nd negativity: retinal ganglion cells [27]	N1: for comparison with the other method P1^12^, 1st positivity: retinal ganglion cell bodies [18] N2^12^, 2nd negativity: optic nerve head [18]
Reporting	Individual waves and PhNR ratio^13^: N1, P1, and N2 wave, both summed across 2 eccentricities and whole stimulated field	N1, P1 and N2 summed across 4 eccentricities and whole stimulated field

^1^further details [14, 17]:

^2^further details [19]:

^3^VERIS 5.1.12XScience (EDI: Electro-Diagnostic Imaging, Redwood City, CA, USA)

^4^MDG403, Philips; P45 phosphor

^5^contrast-inverted version of pattern 1; pattern reversal is extracted by extracting the 2nd order kernel

^6^Grass Model 12, Astro-Med, Inc., West Warwick, RI, USA)

^7^analysis with Igor (IGOR Pro, WaveMetrics, Portland)

^8^Option of re-recording each segment if artefacts like blinking or eye movement occurred

^9^dilation to at least 7 mm with tropicamide 0.5% (Mydriaticum Stulln^®^ UD, Pharma Stulln GmbH, Germany) and phenylephrine hydrochloride 5% (Neo-Synephrine-POS, URSAPHARM Arzneimittel GmbH, Germany)

^10^further details: [23]; the signal to noise ratio (SNR) is defined reflecting the magnitude of the responses [28] and is calculated as the ratio of the root-mean-square (RMS) of the signal and noise windows. SNRs are reported after being logarithmized (logSNR) to follow a normal distribution

^11^“photoreceptors/bipolars” will be used to describe the two generators of the mfERG_PhNR_-N1 component, i.e. the cone photoreceptors and the bipolar cells [24, 25]

^12^mfPERG-P1 and -N2 resemble transient PERG-P50 and -N95, but are subject to higher stimulation rate

^13^ PhNR ratio is calculated based on the findings of a previous study [17] and ISCEV recommendations [29], i.e., to determine whether N2 reduction is due to proximal or distal generators

mfERG_PhNR_: multifocal electroretinogram to record photopic negative response; mfPERG: multifocal pattern electroretinogram; VF: visual field

^†^N2 is also called photopic negative response (PhNR) of mfERG_PhNR_

For better compliance with DTL electrodes, a local eye anaesthetic (Conjucain^®^ EDO^®^ 0.4 mg/1 ml, agent: Oxybuprocainhydrochlorid 2 mg/ml) was given. Further details about stimulation and recording are summarized in Table 1, following Al-Nosairy et al. [20].

### Statistical analysis

Both eyes of each participant were analyzed to reveal any interocular differences. To depict visual fields of ERG from both eyes, the traces from right eyes were left–right flipped.

After extraction of the mfPERG and mfERG_PhNR_ parameters given in Table 1, the data were exported from IGOR to SPSS 26 (Statistical Package for the Social Sciences; IBM, Armonk, NY, USA) and R [30] for further analysis. Data were presented as mean ± SD when testing of Shapiro–Wilk test for normality was not rejected. Four-way repeated measures ANOVAs (RM-ANOVA) were applied to investigate the influence of the factors ECCENTRICITY, AGE, EYE and ELECTRODE for mfPERG recording. For the mfERG_PhNR_, DILATATION was additionally included in the analysis, a five-way RM-ANOVA was conducted. SNR were logarithmized (logSNR) for statistical analyses to follow a normal distribution [28]. Post-hoc testing, if applicable, was performed with p-value corrections after Sidak for multiple comparisons [31].

Interocular ERG asymmetry was calculated as follows:

$${\text{asymmetry}}\left[ \% \right] = \left| {{\text{Eye}}_{{{\text{right}}}} - {\text{Eye}}_{{{\text{left}}}} } \right|/\left( {{\text{Eye}}_{{{\text{right}}}} + {\text{Eye}}_{{{\text{left}}}} } \right) \times {1}00.$$

## Results

### E_DTL_ and E_SKIN_ recordings–traces overview

**Fig. 2:**
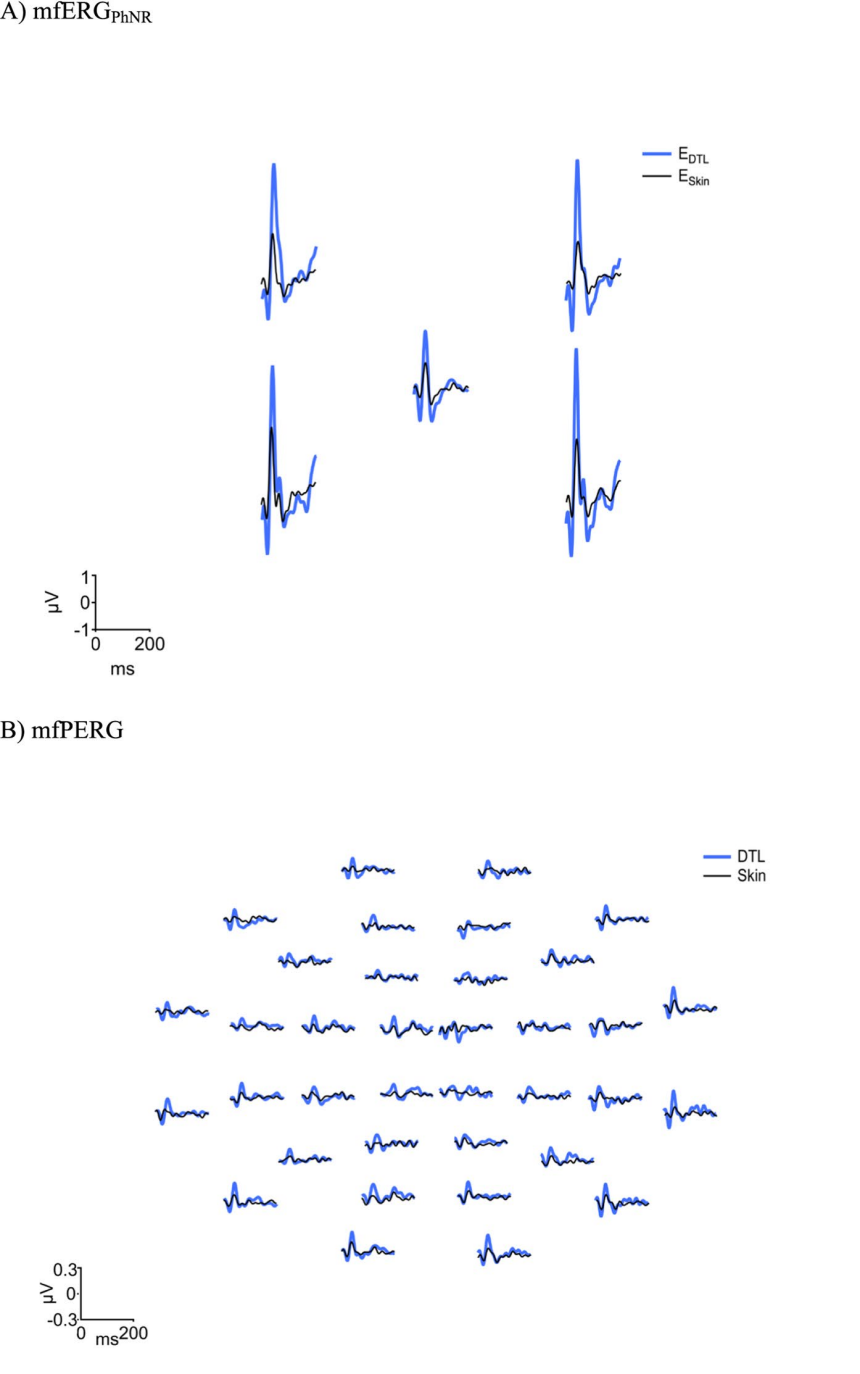
Right eye traces of E_DTL_ and E_SKIN_ of a representative participant. **(A)** mfERG_PhNR_ (dilated) and **(B)** mfPERG recording (non-dilated)

**Fig. 3:**
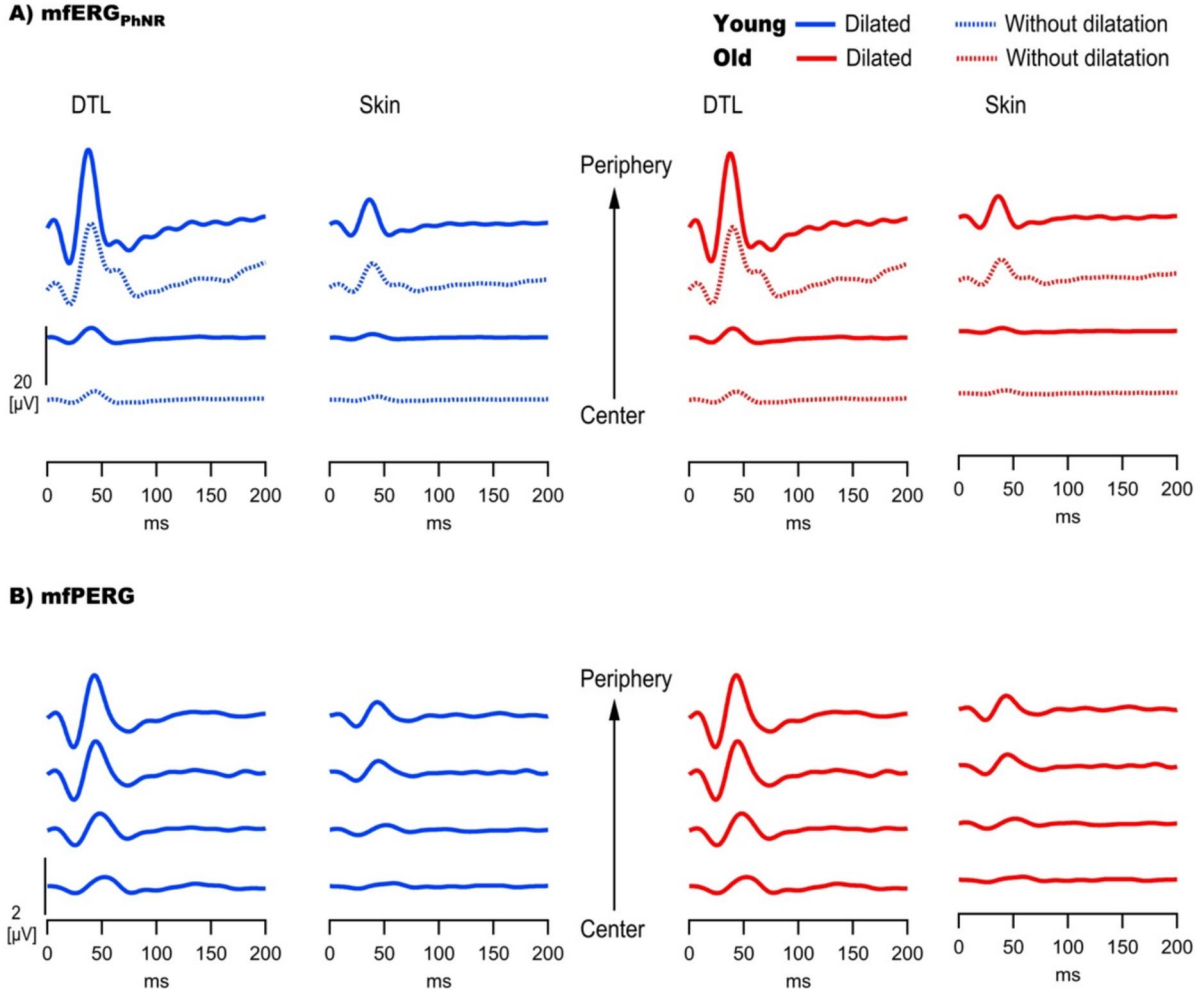
Ring-sum traces across all participants’ right eyes (n = 18) for E_DTL_ and E_SKIN_ for **(A)** mfERG_PhNR_ and **(B)** mfPERG (dilated). Note higher amplitudes for E_DTL_ and for both electrodes peripherally higher amplitudes and shorter peak times

A qualitative overview of the obtained mfPERG and mfERG_PhNR_ trace arrays of a typical participant is given in Fig. 2 for an initial assessment of the E_DTL_ and E_SKIN_ recordings. An overview over the grand mean ring averages is given in Fig. 3. While the typical features of the recordings, i.e. N1, P1, N2, were evident for both E_DTL_ and E_SKIN_, E_SKIN_ resulted in reduced ERGs. Below we provide a quantitative assessment of the observed effects at the group level.

### mfERG_PhNR_ recordings

**Fig. 4:**
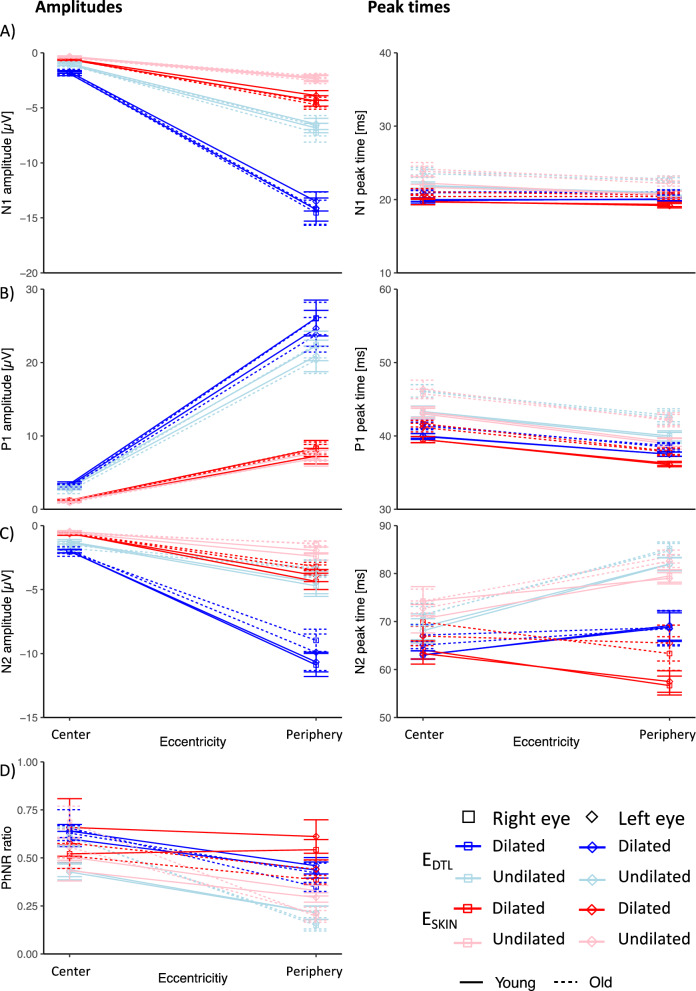
Analysis of mfERG_PhNR_ amplitudes and peak times for **(A)** N1, **(B)** P1, **(C)** N2, **(D)** PhNR ratio across eccentricities for both groups, i.e., Young (*n* = 10) and Old (*n* = 8). For significance levels see text. **(A)** For N1, amplitudes were higher for dilation, E_DTL_ and periphery. Peak times were shorter for Young, dilation, E_SKIN_ and periphery. **(B)** For P1, amplitudes were higher for dilation, E_DTL_ and periphery. Peak times were shorter for Young, dilation, E_SKIN_ and periphery. **(C)** For N2, amplitudes were higher for dilation, E_DTL_ and periphery. Peak times were shorter for dilation and inner eccentricity. **(D)** For PhNR ratio, ratio was higher dilation and centrally

Mean amplitudes, peak times and logSNR were determined for the different components of the mfERG_PhNR_ i.e. for N1, P1, N2 and PhNR ratio, for each individual and averaged within the two groups, i.e. Young and Old. The results for the different conditions (E_SKIN_ & E_DTL_; dilated vs undilated) are depicted in Fig. 4. A 5-way RM-ANOVA was employed to investigate main and interaction effects of i) ELECTRODE ii) DILATATION, iii) EYE, iv) AGE, and v) ECCENTRICITY for amplitude and peak time of each mfERG_PhNR_ component (N1, P1, N2 and PhNR ratio) and for logSNR (Fig. 6A), as detailed below. Interaction effects are given at the end of the section.

(i) Factor ELECTRODE
  *Amplitudes.* In comparison to E_DTL_, E_SKIN_ amplitudes were significantly reduced for all components to 32–38%, except, as expected, for PhNR ratio ($p < 0.001$; E_DTL_ vs E_SKIN_ [µV] mean ± SEM: N1: − 5.9 ± 0.3 vs − 1.9 ± 0.1; P1: 13.2 ± 0.8 vs 4.4 ± 0.4; N2 − 4.4 ± 0.3 vs − 1.7 ± 0.1). *Peak times*. Only for N1 and P1, E_SKIN_ peak times were 0.2 and 0.5 ms earlier than for E_DTL_ ($p < 0.05$ for N1, P1), respectively. *SNR*. E_SKIN_ logSNRs were significantly reduced to 80% ($p < 0.001$, 0.97 E_DTL_ vs 0.78 E_SKIN_).
(ii) Factor DILATATION
  *Amplitudes.* Undilated recordings were significantly reduced for N1, P1, N2 amplitudes and PhNR ratio to 50.5%, 87.2%, 47.2% and 71.4%, respectively, ($p < 0.01$; dilated vs undilated [µV]: N1 − 5.2 ± 0.3 vs − 2.6 ± 0.2, P1 9.7 ± 0.6 vs 8.2 ± 0.5, N2 −4.1 ± 0.3 vs − 1.9 ± 0.2, PhNR ratio [no unit] 0.53 ± 0.03 vs 0.38 ± 0.02). Undilated peak times were delayed by 2.0 ms, 3.7 ms, and 11.8 ($p < 0.001,$ N1, P1, and N2), respectively. *SNR*. Undilated recordings had a minor non-significant trend of a logSNR loss to 96.2%, $p = 0.055$.
(iii) Factor EYE
  *Amplitudes.* We observed a weakly significant minor effect of EYE ($p < 0.05$ for N1 and P1). Following the interocular ERG asymmetry index for N1, P1 and N2 (see methods), amplitudes were comparable by 97.1%, 96.2%, and 99.1%, respectively. No effect of factor EYE was found on PhNR ratio, peak times and logSNR.
(iv) Factor AGE
  *Amplitudes.* We observed no significant effect of AGE on amplitudes and PhNR ratio. *Peak times*. Only for N1 and P1, peak times for Young were 1.5 ms and 2.2 ms, respectively, earlier than for Old ($p = 0.008$ for N1 and $p = 0.011$ for P1). *SNR*. No effect.
(v) Factor ECCENTRICITY
  *Amplitudes.* Central amplitudes were reduced compared to peripheral ones for all components. In contrast, central PhNR ratios were greater than peripheral ones ($p < 0.001$; percentage increase for N1: 86.1%, P1: 86.6%, N2: 76.5% and PhNR ratio: 40.1%). *Peak times*. Central peak times were more delayed than peripheral ones by 0.7, 3.4, and 5.2 ms ($p < 0.001,$ for N1, P1 and N2, respectively). *SNR*. The central logSNR was reduced to 78.9% ($p < 0.001$, 0.78 central vs 0.98 peripheral).
(vi) INTERACTIONS of factors

- DILATATION × ECCENTRICITY $$(p < 0.05)$$
  The delay in central compared to peripheral peak times was only significant in undilated recordings, namely by 1.2 ms for N1 and 10.9 ms for N2 peak times ($p < 0.001$). For the PhNR ratio, undilated recordings showed smaller ratios (0.22) only in the periphery (0.46), $p < 0.001$. The DILATATION effect on logSNR was only significant in the central responses of E_DTL_ and E_SKIN_ upon post hoc analysis.
- ELECTRODE × ECCENTRICITY $$(p < 0.001)$$
  For central N1, E_DTL_ (21.6 ms) vs E_SKIN_ (21.6 ms) peak times did not differ, while peripheral ones did (21.2 ms vs 20.7 ms, $p < 0.001$). Same applies for P1 peak times (42.7 ms vs 42.5 ms in central ring, $p > 0.05$, and 39.6 ms vs 38.8 ms in periphery, $p < 0.001$). In contrast, more delayed N2 peak times of E_DTL_ than E_SKIN_ hold both centrally and in the periphery, $p < 0.05$. For N2, more delayed central than peripheral peak times were statistically significant for E_DTL_ (67.3 vs 76 ms, $p < 0.001$) in contrast to comparable peak times for E_SKIN_ (69.5 vs 71 ms, $p > 0.05$). For PhNR ratio, E_DTL_ vs E_SKIN_ ratios did not differ in the center (0.58 vs 0.56, $p > 0.05$), but in the periphery (0.30 vs 0.38, $p = 0.001$).
- ELECTRODE × ECCENTRICITY × DILATATION ($p < 0.05$)
  For N2, in the central ring E_DTL_ dilated amplitudes were not greater than undilated E_SKIN_ amplitudes (0.076 µV difference, $p > 0.05$).

Taken together, for all mfERG_PhNR_ components we observed higher amplitudes for E_DTL_, dilated recordings and periphery than for E_SKIN_, undilated recording, and center, respectively. PhNR ratios were higher for dilated recordings and inner eccentricities. Peak times were shorter for E_SKIN_, dilated recordings and periphery. It should be noted that the peak time delay for E_DTL_ vs E_SKIN_ depended on eccentricity for N1, P1 and N2. Specifically, for N1 and P1 the E_DTL_ delay applied to the periphery only. For Young compared to Old, peak times were earlier independent of electrode (Table 2).

**Table 2:** mfERG_PhNR_ amplitudes and peak times: F value, df, n^2^ effect size, *p*-values if $p < 0.05$

Factor/Interaction	N1 amp	N1 peak times
AGE		$f\left( {1,16} \right) = 9.16,{ }\eta^{2} = 0.36,{ }p = 0.008{ }$
DILATATION	$f\left( {1,16} \right) = 137.76,\,\upeta^{2} = 0.90,{ }p < 0.001{ }$	$f\left( {1,16} \right) = 112.55,\,\upeta^{2} = 0.88,{ }p < 0.001{ }$
ECCENTRICITY	$f\left( {1,16} \right) = 449.90,\,\upeta^{2} = 0.97,{ }p < 0.001{ }$	$f\left( {1,16} \right) = 40.69,\,\upeta^{2} = 0.72,{ }p < 0.001{ }$
ELECTRODE	$f\left( {1,16} \right) = 225.71,\,\upeta^{2} = 0.93,{ }p < 0.001{ }$	$f\left( {1,16} \right) = 6.84,\,\upeta^{2} = 0.30,{ }p = 0.019{ }$
EYE	$f\left( {1,16} \right) = 4.67,\,\upeta^{2} = 0.23,{ }p = 0.046{ }$	
Dil^1^ × Ecc^1^	$f\left( {1,16} \right) = 193.56,\,\upeta^{2} = 0.92,{ }p < 0.001{ }$	$f\left( {1,16} \right) = 21.99,\,\upeta^{2} = 0.58,{ }p < 0.001$
Dil × El^1^	$f\left( {1,16} \right) = 109.85,\,\upeta^{2} = 0.87,{ }p < 0.001{ }$	$f\left( {1,16} \right) = 6.72,\,\upeta^{2} = 0.30,{ }p = 0.020$
El × Ecc	$f\left( {1,16} \right) = 232.03,\,\upeta^{2} = 0.94,{ }p < 0.001{ }$	$f\left( {1,16} \right) = 15.85,\,\upeta^{2} = 0.50,{ }p = 0.001$
Dil × El × Ecc	$f\left( {1,16} \right) = 116.73,\,\upeta^{2} = 0.88,{ }p < 0.001{ }$	

	P1 amp	P1 peak times
AGE		$f\left( {1,16} \right) = 8.24\,\upeta^{2} = 0.34,{ }p = 0.011$
DILATATION	$f\left( {1,16} \right) = 11.97,\,\upeta^{2} = 0.43,{ }p = 0.003$	$f\left( {1,16} \right) = 103.62,\,\upeta^{2} = 0.87,{ }p < 0.001{ }$
ECCENTRICITY	$f\left( {1,16} \right) = 246.80,\,\upeta^{2} = 0.94,{ }p < 0.001{ }$	$f\left( {1,16} \right) = 273.53,\,\upeta^{2} = 0.95,{ }p < 0.001$
ELECTRODE	$f\left( {1,16} \right) = 225.83,\,\upeta^{2} = 0.93,{ }p < 0.001$	$f\left( {1,16} \right) = 28.19,\,\upeta^{2} = 0.64,{ }p < 0.001$
EYE	$f\left( {1,16} \right) = 7.56,\,\upeta^{2} = 0.32,{ }p = 0.014$	
Dil × Ecc	$f\left( {1,16} \right) = 11.82,\,\upeta^{2} = 0.43,{ }p = 0.003$	$f\left( {1,16} \right) = 13.91,\,\upeta^{2} = 0.47,{ }p = 0.002$
Dil × El	$f\left( {1,16} \right) = 11.82,\,\upeta^{2} = 0.43,{ }p = 0.003$	$f\left( {1,16} \right) = 7.17,\,\upeta^{2} = 0.31,{ }p = 0.016$
El × Ecc	$f\left( {1,16} \right) = 217.67,\,\upeta^{2} = 0.93,{ }p < 0.001$	$f\left( {1,16} \right) = 49.02,\,\upeta^{2} = 0.75,{ }p < 0.001$
EYE × Ecc	$f\left( {1,16} \right) = 6.92,\,\upeta^{2} = 0.30,{ }p = 0.018$	
Dil × El × Ecc	$f\left( {1,16} \right) = 13.51,\,\upeta^{2} = 0.46,{ }p = 0.002$	
Dil × El × EYE		$f\left( {1,16} \right) = 4.90,\,\upeta^{2} = 0.24,{ }p = 0.042$

	N2 amp	N2 peak times
DILATATION	$f\left( {1,16} \right) = 63.98,\,\upeta^{2} = 0.80,{ }p < 0.001$	$f\left( {1,16} \right) = 78.92,\,\upeta^{2} = 0.83,{ }p < 0.001{ }$
ECCENTRICITY	$f\left( {1,16} \right) = 172.62,\,\upeta^{2} = 0.92,{ }p < 0.001$	$f\left( {1,16} \right) = 28.66,\,\upeta^{2} = 0.64,{ }p < 0.001{ }$
ELECTRODE	$f\left( {1,16} \right) = 152.46,\,\upeta^{2} = 0.91,{ }p < 0.001$	
Dil × Ecc	$f\left( {1,16} \right) = 76.26,\,\upeta^{2} = 0.83,{ }p < 0.001$	$f\left( {1,16} \right) = 31.41,\,\upeta^{2} = 0.66,{ }p < 0.001{ }$
Dil × El	$f\left( {1,16} \right) = 56.98,\,\upeta^{2} = 0.78,{ }p < 0.001$	$f\left( {1,16} \right) = 8.60,\,\upeta^{2} = 0.35,{ }p = 0.010$
El × Ecc	$f\left( {1,16} \right) = 111.48,\,\upeta^{2} = 0.87,{ }p < 0.001$	$f\left( {1,16} \right) = 26.82,\,\upeta^{2} = 0.63,{ }p < 0.001$
Dil × El × Ecc	$f\left( {1,16} \right) = 50.92,\,\upeta^{2} = 0.76,{ }p < 0.001$	

	SNR	PhNR ratio
DILATATION		$f\left( {1,16} \right) = 24.83,\,\upeta^{2} = 0.61,{ }p < 0.001$
ECCENTRICITY	$f\left( {1,16} \right) = 232.63,\,\upeta^{2} = 0.94,{ }p < 0.001$	$f\left( {1,16} \right) = 70.90,\,\upeta^{2} = 0.82,{ }p < 0.001{ }$
ELECTRODE	$f\left( {1,16} \right) = 41.38,\,\upeta^{2} = 0.72,{ }p < 0.001$	
Dil × Ecc	$f\left( {1,16} \right) = 6.77,\,\upeta^{2} = 0.30,{ }p = 0.019$	$f\left( {1,16} \right) = 22.33,\,\upeta^{2} = 0.58,{ }p < 0.001{ }$
Dil × EYE	$f\left( {1,16} \right) = 4.67,\,\upeta^{2} = 0.23,{ }p = 0.046$	$f\left( {1,16} \right) = 5.40,\,\upeta^{2} = 0.25,{ }p = 0.034{ }$
Ecc × AGE		$f\left( {1,16} \right) = 10.51,\,\upeta^{2} = 0.40,{ }p = 0.005{ }$
El × Ecc	$f\left( {1,16} \right) = 16.19,\,\upeta^{2} = 0.50,{ }p < 0.001$	$f\left( {1,16} \right) = 20.36,\,\upeta^{2} = 0.56,{ }p < 0.001{ }$

^1^Dil: DILATATION, El: ELECTRODE; Ecc: ECCENTRICITY

**Table 3:** mfPERG amplitudes and peak times: F value, df, n^2^ effect size, *p*-values if $p < 0.05$

Factor/Interaction	N1 amp	N1 peak times
AGE		$f\left( {1,16} \right) = 16.80,\,\upeta^{2} = 0.51,{ }p < 0.001$
ECCENTRICITY	$f\left( {1,16} \right) = 35.95,\,\upeta^{2} = 0.89,{ }p < 0.001{ }$	$f\left( {1,16} \right) = 4.48,\,\upeta^{2} = 0.49,{ }p = 0.021$
ELECTRODE	$f\left( {1,16} \right) = 142.23,\,\upeta^{2} = 0.90,{ }p < 0.001{ }$	
Ecc^1^ × AGE		$f\left( {1,16} \right) = 3.55,\,\upeta^{2} = 0.43,{ }p = 0.042$
El^1^ × AGE		$f\left( {1,16} \right) = 16.64,\,\upeta^{2} = 0.51,{ }p < 0.001$
El × Ecc	$f\left( {1,16} \right) = 72.80,\,\upeta^{2} = 0.94,{ }p < 0.001{ }$	
El × EYE × AGE	$f\left( {1,16} \right) = 5.00,\,\upeta^{2} = 0.24,{ }p = 0.04$	$f\left( {1,16} \right) = 4.72,\,\upeta^{2} = 0.23,{ }p = 0.045$

	P1 amp	P1 peak times
AGE		$f\left( {1,16} \right) = 5.90,\,\upeta^{2} = 0.27,{ }p = 0.027$
ECCENTRICITY	$f\left( {1,16} \right) = 39.55,\,\upeta^{2} = 0.89,{ }p < 0.001{ }$	$f\left( {1,16} \right) = 68.17,\,\upeta^{2} = 0.94,{ }p < 0.001{ }$
ELECTRODE	$f\left( {1,16} \right) = 144.14,\,\upeta^{2} = 0.90,{ }p < 0.001{ }$	$f\left( {1,16} \right) = 40.37,\,\upeta^{2} = 0.72,{ }p < 0.001{ }$
El × AGE		$f\left( {1,16} \right) = 4.73,\,\upeta^{2} = 0.23,{ }p = 0.045{ }$
El × Ecc	$f\left( {1,16} \right) = 44.94,\,\upeta^{2} = 0.91,{ }p < 0.001{ }$	$f\left( {1,16} \right) = 9.51,\,\upeta^{2} = 0.67,{ }p = 0.001{ }$

	N2 amp	N2 peak times
AGE		$f\left( {1,16} \right) = 5.13,\,\upeta^{2} = 0.24,{ }p = 0.038$
ECCENTRICITY	$f\left( {1,16} \right) = 47.68,\,\upeta^{2} = 0.91,{ }p < 0.001$	$f\left( {1,16} \right) = 6.55,\,\upeta^{2} = 0.58,{ }p = 0.005{ }$
ELECTRODE	$f\left( {1,16} \right) = 140.56,\,\upeta^{2} = 0.90,{ }p < 0.001$	
El × Ecc	$f\left( {1,16} \right) = 35.28,\,\upeta^{2} = 0.88,{ }p < 0.001$	
EYE × Ecc	$f\left( {1,16} \right) = 3.39,\,\upeta^{2} = 0.42,{ }p = 0.048$	

	SNR
ECCENTRICITY	$f\left( {1,16} \right) = 54.48\,\upeta^{2} = 0.92,{ }p < 0.001$
ELECTRODE	$f\left( {1,16} \right) = 63.36,\,\upeta^{2} = 0.80,{ }p < 0.001$
El × Ecc	$f\left( {1,16} \right) = 15.41,\,\upeta^{2} = 0.77,{ }p < 0.001$

^1El: ELECTRODE; Ecc: ECCENTRICITY^

### mfPERG recordings

**Fig. 5:**
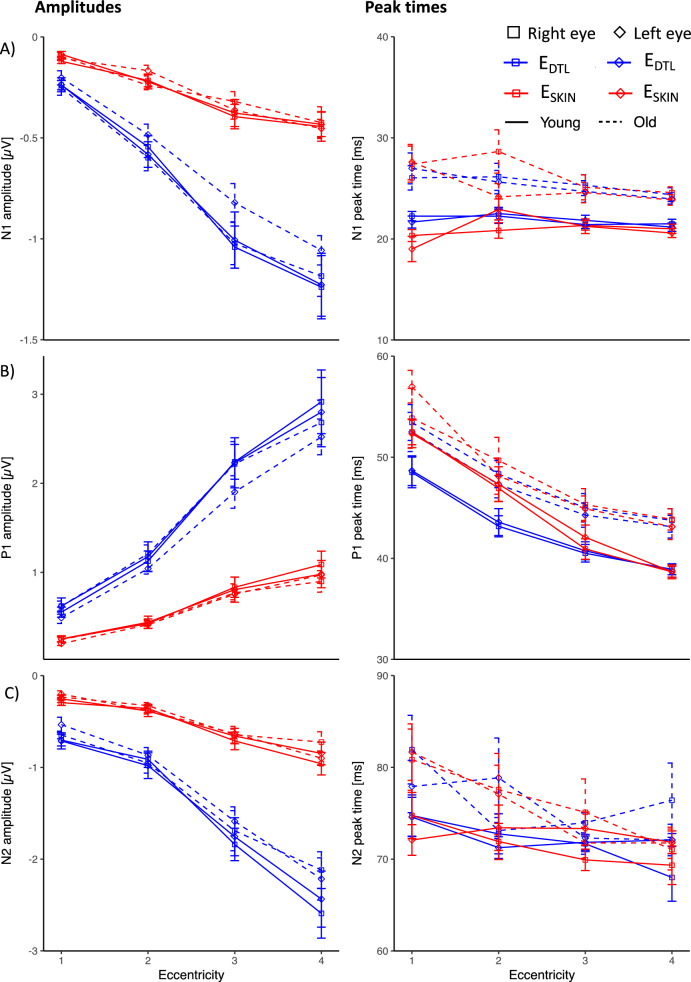
Analysis of mfPERG amplitudes, peak times (**A**) N1 (**B**) P1 (**C**) N2 across different eccentricities for Young (*n* = 10) and Old (*n* = 8). Significance levels of findings can be found in the results. (**A**) For N1, amplitudes were higher for E_DTL_ and periphery. Peak times were shorter for Young and periphery. (**B**) For P1, amplitudes were higher for E_DTL_, periphery and right eye. Peak times were shorter for Young, E_SKIN_ and periphery. (**C**) For N2, amplitudes were higher for E_DTL_, periphery and right eye. Peak times were shorter for Young and periphery

**Fig. 6:**
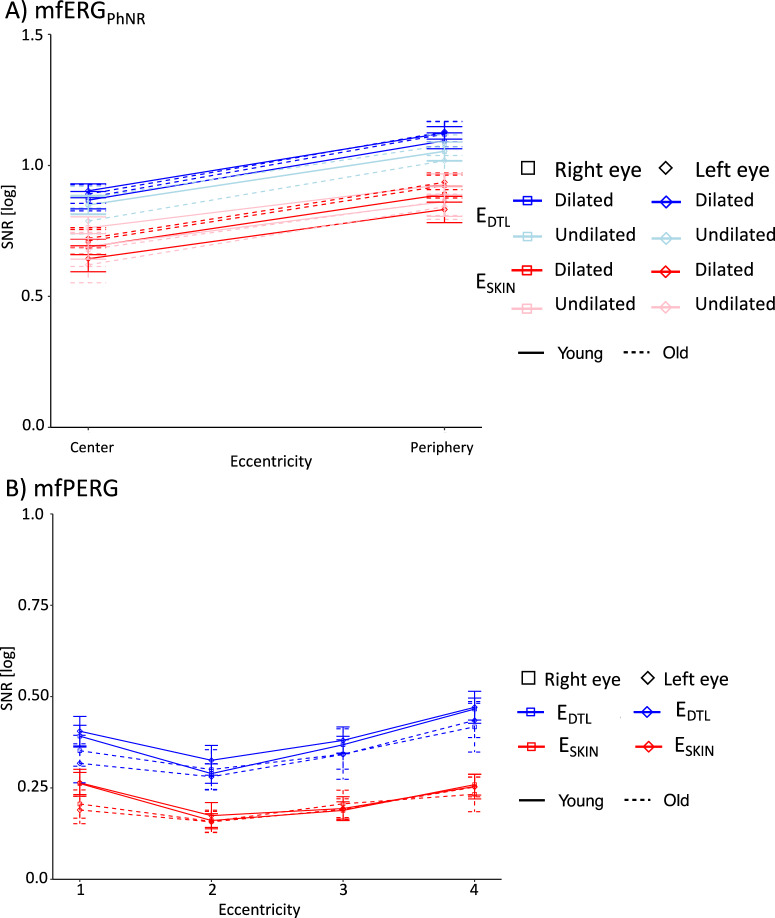
The signal-to-noise ratio of (**A**) mfPERG and (**B**) mfERG_PhNR_. (**A**) LogSNR of mfPERG was higher for E_DTL_ and for eccentricities: r2 < r3 < r1 < r4. (**B**) LogSNR of mfERG_PhNR_ was higher for E_DTL_ and for periphery

Mean amplitude, peak times and logSNR across all participants were determined for different mfPERG components i.e. for N1, P1, and N2, for each individual and averaged within the two groups, i.e. Young and Old (see Figs. 5 and 6B). To investigate main and interaction effects of i) ELECTRODE, ii) EYE, iii) AGE, and iv) ECCENTRICITY, 4-way RM-ANOVA was conducted for each mfPERG component, see Table 3. Interaction effects are given at the end of the section.

(i) Factor ELECTRODE
  *Amplitudes.* E_SKIN_ amplitudes were significantly reduced for all components to 30–38% in comparison to E_DTL_ amplitudes ($p < 0.001$, E_DTL_ vs E_SKIN_ [µV] mean ± SEM: N1 − 0.7 ± 0.1 vs − 0.3 ± 0.02, P1 1.0 ± 0.1 vs 0.5 ± 0.04, N2 − 1.4 ± 0.1 vs − 0.5 ± 0.05). *Peak times*. Only for P1, E_SKIN_ peak times were 1.5 ms shorter than for E_DTL_ ($p = 0.088$ for N1, $p < 0.001$ for P1, $p = 0.995$ for N2). *SNR*. In comparison to logSNR reduction to 80% during mfERG_PhNR_ recording, mfPERG E_SKIN_ logSNRs were significantly reduced to < 60% ($p < 0.001,$ 0.37 E_DTL_ vs 0.21 E_SKIN_), see Fig. 6A.
(ii) Factor EYE
  *Amplitudes*. No effect of factor EYE was found on amplitudes, peak times and SNR. As indicated by the interocular ERG asymmetry index, amplitudes of the left eye were comparable to the right eye for N1, P1, and N2 by 99.4%, 97.5%, and 98.9%, respectively.
(iii) Factor AGE
  *Amplitudes*. We observed no significant effect of AGE on amplitudes. *Peak times*. Young peak times were reduced by 4.2, 3.8, and 3.7 ms compared to Old ($p < 0.05,$ for N1, P1 and N2, respectively). *SNR*. No effect.
(iv) Factor ECCENTRICITY
  *Amplitudes*. The more central the response, the shorter the N1, P1, and N2 amplitude, $p < 0.001$. *Peak times*. mfPERG wavelets showed more delayed central than peripheral peak times, r1 > r2 > r3 > r4: For N1, only r2 (24 ms) showed significant delayed peak times in comparison to r3 (23 ms) and r4 (23 ms), $p < 0.05$. For P1, post hoc tests showed significantly more delayed central than peripheral peak times, r1 52 ms, r2 47 ms, r3 43 ms, and r4 41 ms, $p < 0.001$. For N2, only r1 (77 ms) was more delayed than r3 (72 ms) and r4 (72 ms) peak times, $p < 0.05$. *SNR*. We found logSNRs to generally decrease from center to periphery, $p < 0.001$. This applies for r2 (0.23) > r3 (0.28) > r4 (0.35), $p < 0.001$. LogSNR of r1 (0.30) is, however, significantly greater than r2, $p < 0.001$.
(v) INTERACTIONS of factors

- AGE × ELECTRODE ($$p < 0.001$$)
  For N1 peak time in E_SKIN_ recordings, only Young had shorter peak times by 0.9 ms.
- ELECTRODE × ECCENTRICITY ($$p < 0.005$$)
  LogSNRs showed this interaction due to E_DTL_ logSNR of r1 (0.37) being significantly different from r2 (0.3) and r4 (0.45), $p < 0.01$, in comparison to E_SKIN_ logSNR r1 (0.23) being signficant difference from r2 (0.16), $p < 0.01$.

Taken together, amplitudes were higher for E_DTL_ and for peripheral responses for both electrodes. P1 peak times were earlier for E_SKIN_ than for E_DTL_. It should be noted that differences in logSNR for E_DTL_ vs E_SKIN_ differed for eccentricities (E_DTL_: r1 0.37, r2 0.3; E_SKIN_: r1 0.23, r2 0.16). For Young compared to Old, peak times were earlier independent of electrode.

## Discussion

E_SKIN_ mfERG_PhNR_ [mfPERG] recordings had lower amplitudes of 32–38% [37–38%] and a 80% [60%] of logSNR in comparison to E_DTL_ recordings. Peak times were shorter by 0.4–3.3 ms [0.004–1.3 ms], respectively. Young, while having amplitudes and logSNR comparable to Old, had shorter peak times by 1.5–2.2 ms [3.7–4.2 ms] in either electrode. Both techniques showed good interocular symmetry of 97.1%, 96.2%, and 99.1% [99.4%, 97.5%, and 98.9%] for N1, P1, and N2, respectively, without any electrode effect. In comparison to dilated mfERG_PhNR_ recordings, the undilated responses showed amplitude reductions by 13–53% and delayed peak times by 2.0–11.8 ms.

*Electrode effect* The main goal of this study was to address an unmet need in clinical electrophysiology, i.e. the utility of E_SKIN_ in multifocal ERG recordings, specifically mfERG_PhNR_ and mfPERG. In line with our findings of an amplitude reduction to 32–38% and 37–38%, respectively, Eckermann et. al. reported a reduction to 29% for standard mfERG responses [21]. This is also comparable to conventional ERG studies, i.e., 40% for PhNR [10], 73% for PERG [11], 25% for ffERG [6] and 26–57% for flash ERG [2]. It seems that the consensual finding of reduced amplitudes might be related to the greater distance between potential generators or between eye/lid gap and the recording site [32, 33]. As far as the SNR is concerned when comparing electrodes, previous studies also demonstrated for E_SKIN_ a reduction to 62% and to 40% for PhNR [8] and mfERG SNRs [21], respectively, in line with our study (reduction to 60–80%). The SNR loss for E_SKIN_ recordings might be related to E_SKIN_’s higher impedances and stronger influence of muscle activities, e.g. of the eye lid, than E_DTL_ [7].

Compared to amplitudes, the effect of E_SKIN_ on peak times was more heterogenous. Our observations showed shorter peak times of 0.4 ms in N1 and 0.8 ms in P1 of mfERG_PhNR_ and 1.5 ms in P1 of mfPERG, predominantly for peripheral locations. In line with that, Eckermann et al. [21] reported for mfERG P1-peak times to be reduced by 1.5 ms for E_SKIN_. Studies on ERG_PhNR_ reported shorter peak times by 0.8 ms for a- and b-wave and prolonged peak times of 1.2 ms for PhNR [8]. Others found comparable peak times [10]. For flashERG, shorter peak times of an average of 1.3 ms were found [2] and 6.1 ms shorter peak times for a-wave and 4.2 ms prolonged times for b-wave using gold foil electrodes compared to Burian-Allen electrodes in ffERG in adults [4]. While we do not have an exact explanation for shorter peak times for E_SKIN_, it could be due to the spatial distribution and orientation of the retinal generator sites of each ERG method [8]. Interestingly, for the mfERG recordings, we found central peak time delays for E_DTL_ compared to E_SKIN_ only for N2, but not for N1 and P2. This might indicate that the N2 generators are differently tapped by the two electrode types used. The relevance and potential clinical applications of this observation need to be addressed in future studies.

*Dilatation effect* Another confound factor which might influence E_SKIN_ and E_DTL_ recordings is dilatation which is the standard procedure for mfERG_PhNR_ recording. We found amplitudes to be reduced to 50%, 87%, and 47% for N1, P1, and N2, respectively. In terms of peak times, our study reported 3.7 ms shorter P1 peak times for dilated pupils. In accordance, Schimitzek and Bach suggested that the amplitude in mfERG rose by 20% and peak times decreased by less than 1.5 ms when increasing the luminance by a factor of 3.3 [34], which supports the correlation between brightness and amplitude level. Mohamad-Rafiuddin et al. reported for mfERG that the effect of dilation was not significant in the central retina, more precisely in ring 1 and ring 3, for N1 and P1 [35]. In line with our findings, a reduction to 86% of P1 amplitude of ffERG with undilated pupils has previously been reported [4].

The findings of higher amplitudes and shorter peak times with dilated recordings, might be explained by higher illumination of the retina via dilated pupils [36]. The mfERG amplitudes and peak times appeared to be equal in undilated eyes when setting the luminance five times higher [37]. Dilatation in itself does not influence the differences between E_DTL_ and E_SKIN_, which indicates the possibility to record with both electrodes undilated, if required, but at the expense of reduced amplitudes and increased peak times. Naturally, appropriate reference data, i.e. undilated, would ideally be required.

*Age effect* In both electrodes, the factor AGE did not show any significant influence on the amplitudes of mfERG_PhNR_ and mfPERG. This is consistent with some previous studies that reported no effect of age on N1, P1 and N2 amplitude in mfERG_PhNR_ [38] and in mfPERG [39], while other authors reported an amplitude decrease of 4 to 7.8% per decade in mfERG [40]. Regarding peak times, we did find an effect of age which was independent of electrode on N1 and P1 in mfERG_PhNR_ and N1, P1, and N2 in mfPERG. Kurtenbach et al. reported for mfERG no age effect on N1 and N2 peak times, but for P1 peak time [39]. Both electrodes seem to be sensitive enough to pick up group differences, here demonstrated for the response delay in elderly.

*Eye effect* Interocular differences are an important consideration when assessing asymmetric pathologies of retina and optic nerve head. In our study, both electrodes picked up comparable responses from both eyes with an interocular asymmetry of ≤ 8% in mfERG_PhNR_ and mfPERG. Some authors found no significant differences between eyes for E_DTL_ and E_SKIN_ using ERG_PhNR_ [10], while others do. Interocular differences of 11–14% in b-wave for ffERG were shown [41] and 12% interocular asymmetry for PERG [42]. Another mfERG study found an 18.7% interocular asymmetry of amplitudes under binocular recording settings and 21.6% under monocular conditions [43]. As a reference, differences in amplitudes between eyes are considered significant for ERG_PhNR_ if they are above 50% [10] and for PERG if they are above 30.5% [44]. In short, comparable eye responses were picked by both E_SKIN_ and E_DTL_ in both mfERG_PhNR_ and mfPERG that are within the range of the cited literature for interocular asymmetry.

*Eccentricity effect* We found mfERG_PhNR_ and mfPERG amplitudes to be smaller and peak times to be more delayed, the more central the response. While logSNR was higher in the periphery for mfERG_PhNR_, it was lower in the periphery for mfPERG. Eckermann et al. found the components of mfERG to correlate strongly and significantly across all eccentricities between E_DTL_ and gold cup electrodes [21] with a tendency of higher amplitudes in inner eccentricities and shorter peak times in periphery. Morny et al. using PhNR of ERG found that the response from more eccentric neurons are about three times greater than those in the macular region [45], which supports our findings of higher amplitudes and shorter peak times in peripheral regions. It should be noted, however, we acknowledge that our calculations of amplitudes do not fully reflect another aspect, which is not target of the current study, i.e. the anatomical and physiological aspects of retinal responses. Specifically, we did not consider the size of stimulated area during these calculations, i.e. namely no response density was calculated.

### Clinical relevance

In this study we found E_SKIN_ to produce similar readings of mfERG_PhNR_ and mfPERG compared to E_DTL_ readings. However, for E_SKIN_ amplitudes and SNR are clearly reduced and a peak time reduction is observed for most components. This leads to potential limitations which must be considered in clinical and research applications of E_SKIN._ With respect the peak time effects, it should be noted that some were clearly in the sub-millisecond range, questioning their clinical relevance in addition to sampling limitations. The later refers to the discrete sampling intervals of 0.833 ms which represent a limitation to the precision of the peak time measurements. Moreover, the use of E_SKIN_ might, with respect of participant comfort, also have disadvantages, as previous studies reported uncomfortable and irritating experience of some participants during skin preparations [3, 11, 21]. With respect to dilation for mfERG_PhNR_ recordings, we suggest that in cases of intolerance to dilatation eye drops, i.e., with a narrow chamber angle, both electrodes could be used to record mfERG_PhNR_ at the expense of lower amplitudes and more delayed peak times, ideally with appropriate reference data.

## Conclusion

E_SKIN_ provided plausible mfERG_PhNR_ and mfPERG recordings, but with smaller amplitudes, lower SNRs and shorter peak times compared to E_DTL_ recordings. For age effects, both ERG methods had greater peak times in elderly. For mfERG_PhNR,_ undilated recording was plausible in comparison to dilated recording with trade-off their lower amplitudes and more delayed peak times.

## Declarations

### Statement on the welfare of animals

This article does not contain any procedures with animals.
